# Protein-protein interactions underlying the behavioral and psychological symptoms of dementia (BPSD) and Alzheimer’s disease

**DOI:** 10.1371/journal.pone.0226021

**Published:** 2020-01-17

**Authors:** Yimin Mao, Daniel W. Fisher, Shuxing Yang, Rachel M. Keszycki, Hongxin Dong

**Affiliations:** 1 School of Information and Technology, Jiangxi University of Science and Technology, Jiangxi, China; 2 Applied Science Institute, Jiangxi University of Science and Technology, Jiangxi, China; 3 Department of Psychiatry and Behavioral Sciences, Feinberg School of Medicine, Northwestern University, Chicago, Illinois, United States of America; Universitat Pompeu Fabra, SPAIN

## Abstract

Alzheimer’s Disease (AD) is a devastating neurodegenerative disorder currently affecting 45 million people worldwide, ranking as the 6^th^ highest cause of death. Throughout the development and progression of AD, over 90% of patients display behavioral and psychological symptoms of dementia (BPSD), with some of these symptoms occurring before memory deficits and therefore serving as potential early predictors of AD-related cognitive decline. However, the biochemical links between AD and BPSD are not known. In this study, we explored the molecular interactions between AD and BPSD using protein-protein interaction (PPI) networks built from OMIM (Online Mendelian Inheritance in Man) genes that were related to AD and two distinct BPSD domains, the Affective Domain and the Hyperactivity, Impulsivity, Disinhibition, and Aggression (HIDA) Domain. Our results yielded 8 unique proteins for the Affective Domain (RHOA, GRB2, PIK3R1, HSPA4, HSP90AA1, GSK3beta, PRKCZ, and FYN), 5 unique proteins for the HIDA Domain (LRP1, EGFR, YWHAB, SUMO1, and EGR1), and 6 shared proteins between both BPSD domains (APP, UBC, ELAV1, YWHAZ, YWHAE, and SRC) and AD. These proteins might suggest specific targets and pathways that are involved in the pathogenesis of these BPSD domains in AD.

## Introduction

Alzheimer’s Disease (AD) is a progressive neurodegenerative disease and is the most common form of dementia with over 40 million people affected worldwide[1]. Surprisingly, over 90% of AD patients display behavioral and psychological symptoms of dementia (BPSD), including agitation, aggression, irritability, impulsivity, disinhibition, anxiety, depression, apathy, euphoria, and psychosis[2, 3]. BPSD can present at almost any stage of AD, and in some patients, these symptoms can even appear before memory deficits develop[4]. The severity of BPSD increases significantly with disease progression, and BPSD affect the quality of life of both patients and their caregivers[5–7]. Though memory deficits are the best studied aspects of AD, it is BPSD that are often the greatest source of burden for patients and caregivers and are one of the main reasons for institutionalization[8–11]. Along with there being few rigorous studies of BPSD’s biochemical and cellular mechanisms, there are no FDA-approved treatments for BPSD management[12, 13].

Although BPSD present differently in each patient, the presence of certain symptoms makes the co-occurrence of other symptoms more likely. Thus, it has been suggested that certain symptoms cluster into behavioral domains and that these domains may have commonly disturbed molecular pathways at their core, explaining the higher likelihood of certain symptoms presenting together[2]. Among the myriad of BPSD, unbiased clustering approaches generally yield the following 5 domains: Affective Domain; Hyperactivtiy, Impulsivity, Disinhibition, Aggression (HIDA) Domain; Apathy Domain; Psychosis Domain; and Euphoria Domain [2]. However, the distinct molecular mechanisms leading to the common presentation of symptoms in each domain and how AD pathogenesis leads to these molecular alterations is surprisingly unstudied.

Recently, investigation of the interactions between proteins encoded in known disease genes through protein-protein interaction (PPI) networks has become a powerful approach to exploring the etiology and neuropathology of complex diseases, including AD[14, 15]. PPI data can be used at a larger scale to map networks of interactions depending on their functional associations[16]. Research studies based on PPI networks have achieved noteworthy results, revealing disease complexities at the protein and gene levels[14, 15, 17–23]. Among them, some studies[14, 15, 17, 24] have used PPI to discover essential proteins, genes, and associated pathways linked to disease pathogenesis and potential therapies. For example, using such methods, researchers have identified candidate genes[14] and signaling pathways involved in AD pathogenesis in a brain region-specific manner[15]. However, the proteomic links between AD and BPSD have not been investigated.

In this study, using the PPI network analysis approach, we investigated the proteomic links between AD and select BPSD domains, namely the Affective Domain and HIDA Domain. We selected these two BPSD domains as their symptoms are the best studied outside AD pathogenesis. We first chose causative genes related to these symptoms based on prior information from the Online Mendelian Inheritance in Man (OMIM) database and published literature, then we constructed PPI networks related to AD and the two BPSD domains. After, we designed a DBruteForce algorithm to detect shared proteins. Finally, based on the “centrality-lethality” paradigm[25], we designed a “shared protein-degree centrality” principle to identify essential shared proteins between AD and each BPSD domain. Our study reveals intrinsic protein connections between AD and BPSD.

## Materials and methods

**Fig 1:**
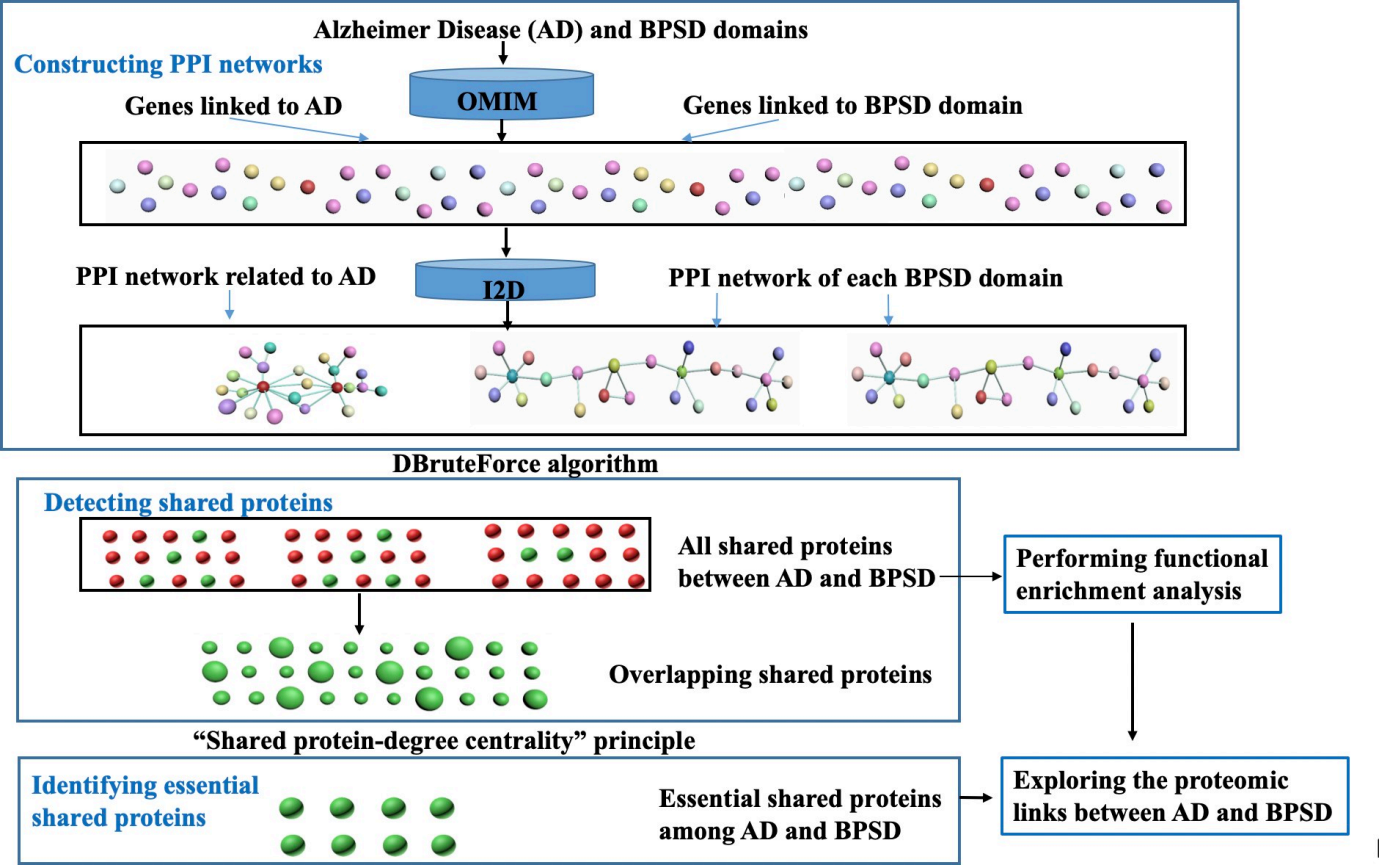
The overall study workflow used to explore the proteomic links between AD and BPSD symptoms. The input terms were identified using the OMIM database and the PPI networks were constructed using information from I2D. The DBruteForce algorithm was used to detect shared proteins and then the “shared protein-degree centrality” principle was applied to identify the essential shared proteins between AD and each BPSD domain. The enrichment analyses were done on the shared proteins that were detected after using the DBruteForce algorithm. Red color indicates the shared proteins between AD with one BPSD domain, and the green color indicates the shared proteins among AD with both domains.

The analytical framework to identify the shared essential proteins is illustrated schematically in **Fig 1.** The process consists of three main steps–Construction, Detection, and Identification: 1) Construction involves building the PPI networks from the Interologous Interaction Database (I2D); 2) Detection involves investigating the shared proteins between diseases by designing a DBruteForce algorithm; and 3) Identification involves searching essential shared proteins by designing a “shared protein-degree centrality” principle.

### Databases

Two databases: OMIM and I2D were used in this study. OMIM is classical database containing an authoritative compendium of human genotypes and associated phenotypes and contains over 15,000 genes. I2D is an online database of known and predicted mammalian and eukaryotic protein-protein interactions. It has integrated known, experimental, and predicted PPIs for five model organisms and humans. I2D is comprised of more than 687,000 experimental PPIs and about 619,000 predicted interactions. Notably, two points make I2D preferred over other interaction databases: 1) it has been built by mapping high-throughput data between species, with these interactions being considered "*predictions"*; 2) it remains one of the most comprehensive sources of known and predicted eukaryotic PPIs. The most recent updates for the databases we used were in April 2019.

### Identification of input genes relating to AD and BPSD

First, we selected symptom terms that matched our AD and BPSD Domains, including “Alzheimer’s Disease”, “anxiety”, “depression”, “MDD”, “hyperactivity”, “disinhibition”, “impulsivity” and “aggression.” Then, we input these terms into the OMIM database and reviewed the list of hereditary disease genes from the OMIM morbid map (http://www.omim.org). As these search terms are broad, many of the resulting genes identified in OMIM are not clearly associated with these symptoms. For instance, the search term ‘hyperactivity’ can bring up genes involved in both aberrant motor behavior as well as an increased ability of cells to fire an action potential. Thus, two investigators independently determined which potential genes were truly implicated in causing each symptom based on the OMIM report and published literature. Agreement between the investigators was >90%, and a third investigator determined if a gene should be added in the case of a disagreement.

### Construction of PPI networks

The “causative proteins” for AD, the Affective Domain, and the HIDA Domain from OMIM were input to the I2D database (http://ophid.utoronto.ca/ophidv2.204/ppi.jsp) with ‘human’ as the chosen target organism, and the resultant PPIs related to AD and BPSD were generated, including predicted and experimental PPIs. To increase the data reliability of protein interactions, all predicted homologous protein interactions were excluded. The interaction network contained the disease-associated proteins (nodes) and their interactions (edges). By exploring the mapping scheme of the UniProt database, corresponding gene names and IDs were retrieved. Because some proteins were given multiple names, the results in tables and figures were presented in the format of gene names and UniProt IDs to avoid ambiguous referencing.

### Detection of the shared proteins between AD and BPSD

**Fig 2:**
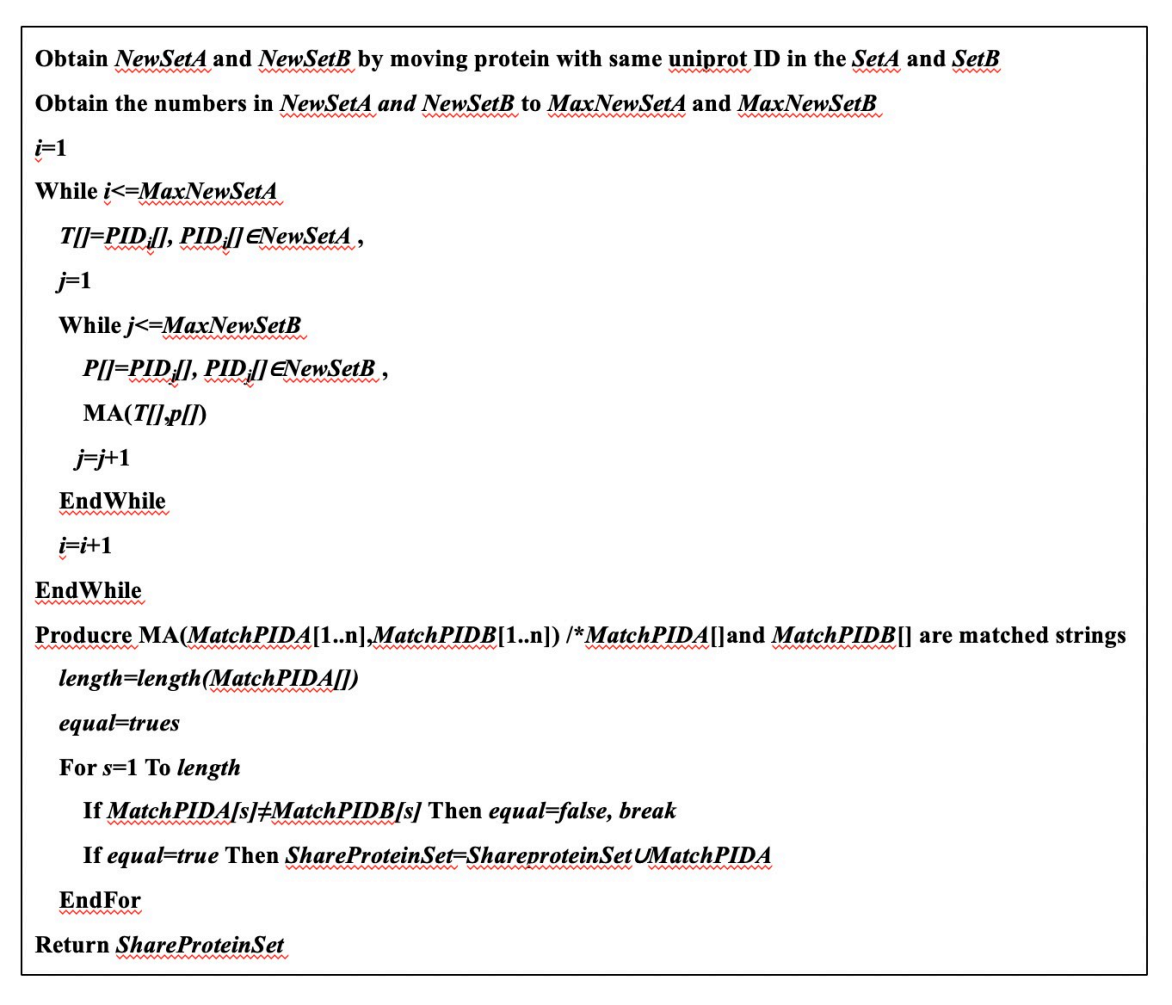
The DBruteForce algorithm.

The connection sets between proteins for AD and the BPSD domains were produced based on the PPI networks constructed for AD or each symptom domain. BruteForce [26] is the simplest string match algorithm for two strings (two Uniprot IDs); however, it us unable to capture intersections between PPI networks. Using this algorithm as an intial framework, we developed Deformation BruteForce algorithm (DBruteForce) to identify the shared proteins between AD and BPSD networks. The DBruteForce algorithm performs intersections of Uniprot IDs for each protein from the AD list with all the proteins in the given BPSD domain list, and these intersection IDs are served as “shared proteins.” Suppose two sets (*setA* and *setB*) of nodes interact between AD and a given BPSD domain produced by the PPI networks. *PID* indicates the Uniprot ID of the protein and is a string. The DBruteForce algorithm used in this study is shown in **Fig 2.**

### Identification of essential shared proteins among AD and BPSD

We identified essential shared proteins between AD and the two BPSD domains based on their node degree centrality. For each node (protein), we applied degree centrality (*DC*) to assess its role in the network. Given a PPI network, it is represented as an undirected graph *G* (*V*, *E*) with proteins as nodes and interactions as edges. Degree centrality is calculated by:

DC(v)=e(u,v)u,v∈V (1)

where *v* represents a node in PPI network, *u* is any node other than *v* in the network, and *e(u*,*v)* represents the interaction between *v* and *u*. If such an interaction exists, the value of *e(u*,*v)* is one. If not, *e(u*,*v)* is zero. *|e(u*,*v)|* represents total interaction numbers between *v* and *u*. According to the “principle of centrality-lethality,” a shared protein with high degree centrality might play an important role in the biological system, thus, it is an essential shared protein[25].

### Functional enrichment analysis

To further study the functions of the proteins in the PPI networks linked to AD and BPSD, an analysis of functional enrichment and enriched pathways was performed with the Gene Ontology (GO) and the Kyoto Encyclopedia of Genes and Genomes (KEGG) databases (Version No DAVID 6.8; https://david.ncifcrf.gov/summary.jsp). In the enrichment analyses, functions or pathways were considered enriched if *P* < .01. To perform these analyses, we annotated, visualized, and integrated discovery by using the DAVID database, which is an online platform providing functional annotation tools to analyze biological meaning behind large gene lists.

## Results

### Genes and interaction networks

**Fig 3:**
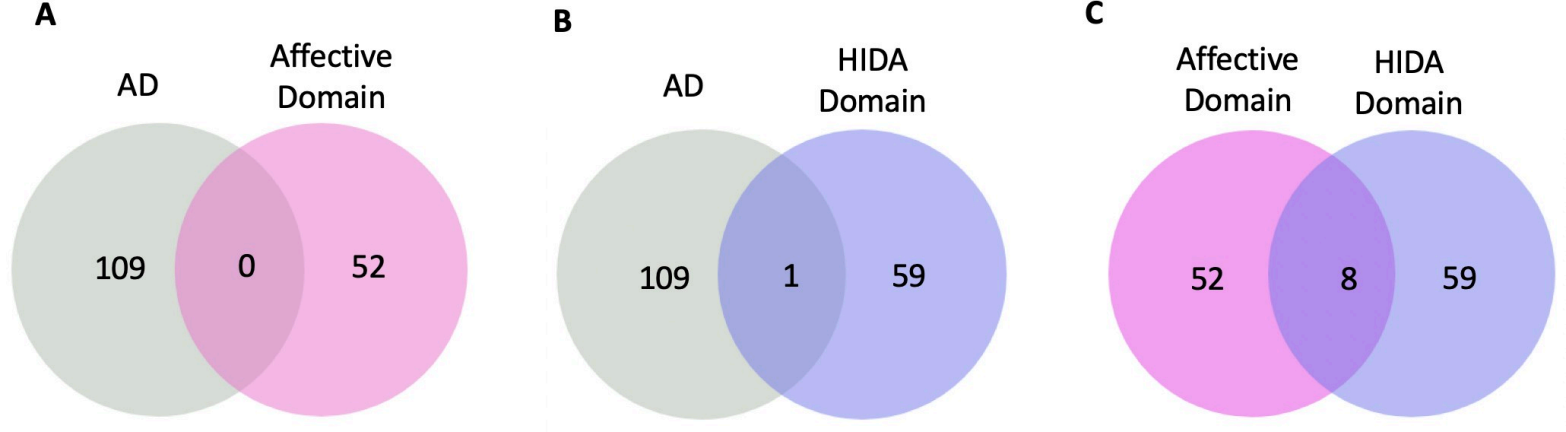
The intersects of genes. A) The intersects of genes between AD and Affective Domain symptoms. B) The intersects of genes between AD and HIDA Domain symptoms. C) The intersects of genes between Affective and HIDA Domain symptoms.

Candidate genes/proteins implicated in the pathogenesis of AD, the symptoms of Affective Domain, and HIDA Domain were obtained from the OMIM database and verified with published literature **(The details are provided in S1 Table)**. The intersects of genes among AD, Affective and HIDA Domains are described in **Fig 3**. There were 0 intersects of genes between AD and Affective Domain, 1 intersect of genes between AD and HIDA Domain, and 8 intersects of genes between Affective and HIDA Domains. The PPI networks comprising these genes/proteins were constructed utilizing information from I2D. The AD-related proteins produced a network with 14,527 nodes, the proteins related to symptoms in Affective Domain produced a network with 4,825 nodes, and the proteins related to symptoms in the HIDA Domain produced a network with 3,127 nodes. After removing the predicted interactions that were homologous to validated interactions, there were 8,747 interactions for AD, 2,763 interaction for the Affective Domain, and 1,754 interactions for the HIDA Domain as well as 3,415 nodes for AD, 2,111 nodes for the Affective Domain and 1,352 nodes for the HIDA Domain.

### Shared proteins between AD and BPSD

**Fig 4:**
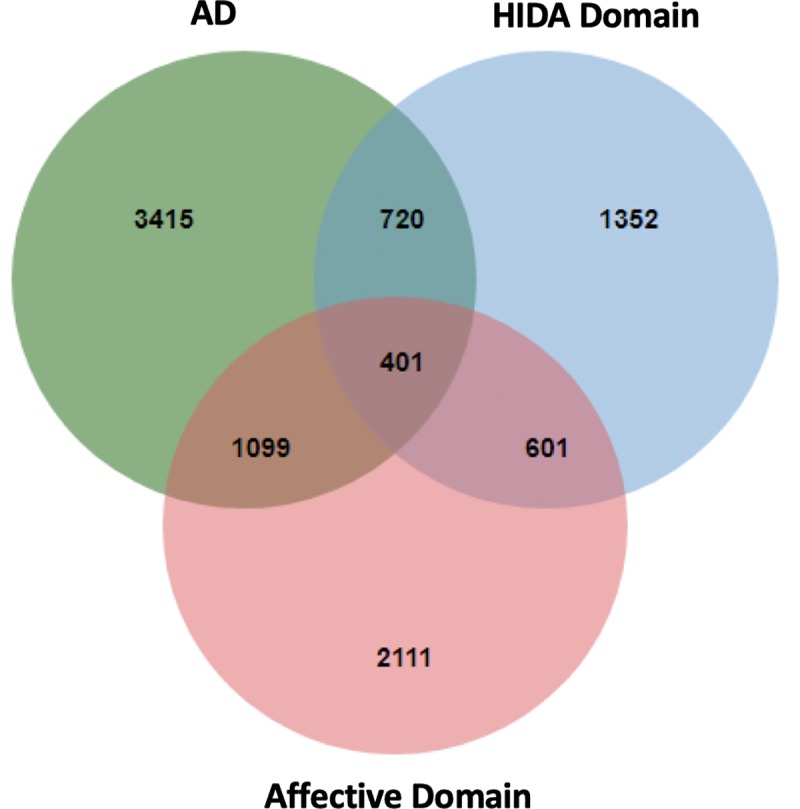
The numbers of shared protein. There were 1099 proteins shared between AD and the Affective Domain, 720 shared between AD and the HIDA Domain, and 401 shared between all three.

The shared proteins between AD and the BPSD domains were detected using a DBruteForce algorithm (**Fig 2**). We found 1,099 shared proteins between AD and the Affective Domains **(S2 Table)**, 720 shared proteins between AD and the HIDA Domain **(S3 Table),** and 401 shared proteins among AD, the Affective Domain, and the HIDA domain **(S4 Table, Fig 4).**

### Essential shared proteins between AD and BPSD

**Fig 5:**
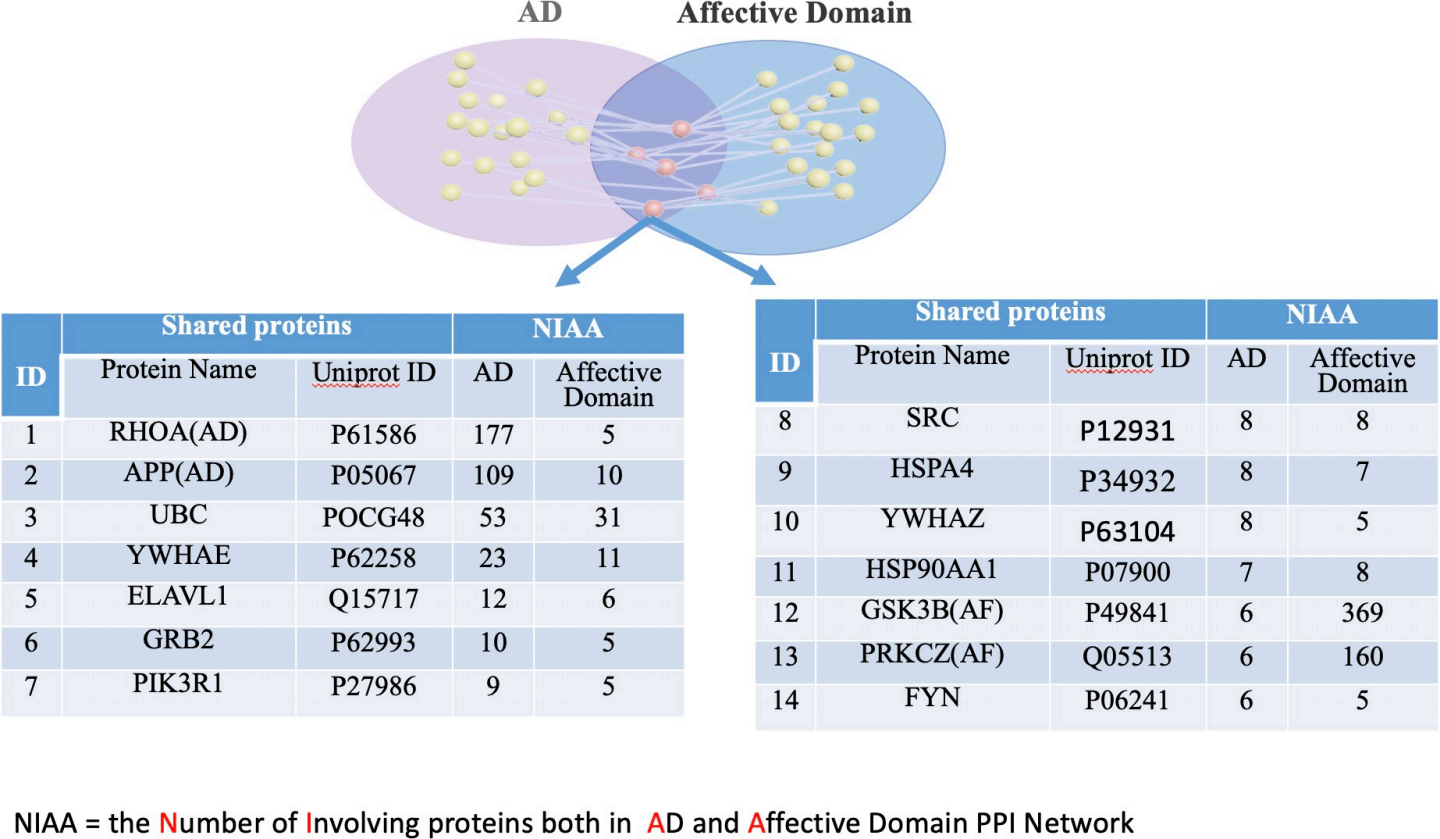
The essential shared proteins between AD and the Affective Domain. There were 14 proteins that were identified as being essential shared proteins between AD and the Affective Domain. Proteins in red are essential shared proteins that are unique to the Affective Domain while those in black were also found to be essential shared proteins between AD, the Affective Domain, and the HIDA Domain.

**Fig 6:**
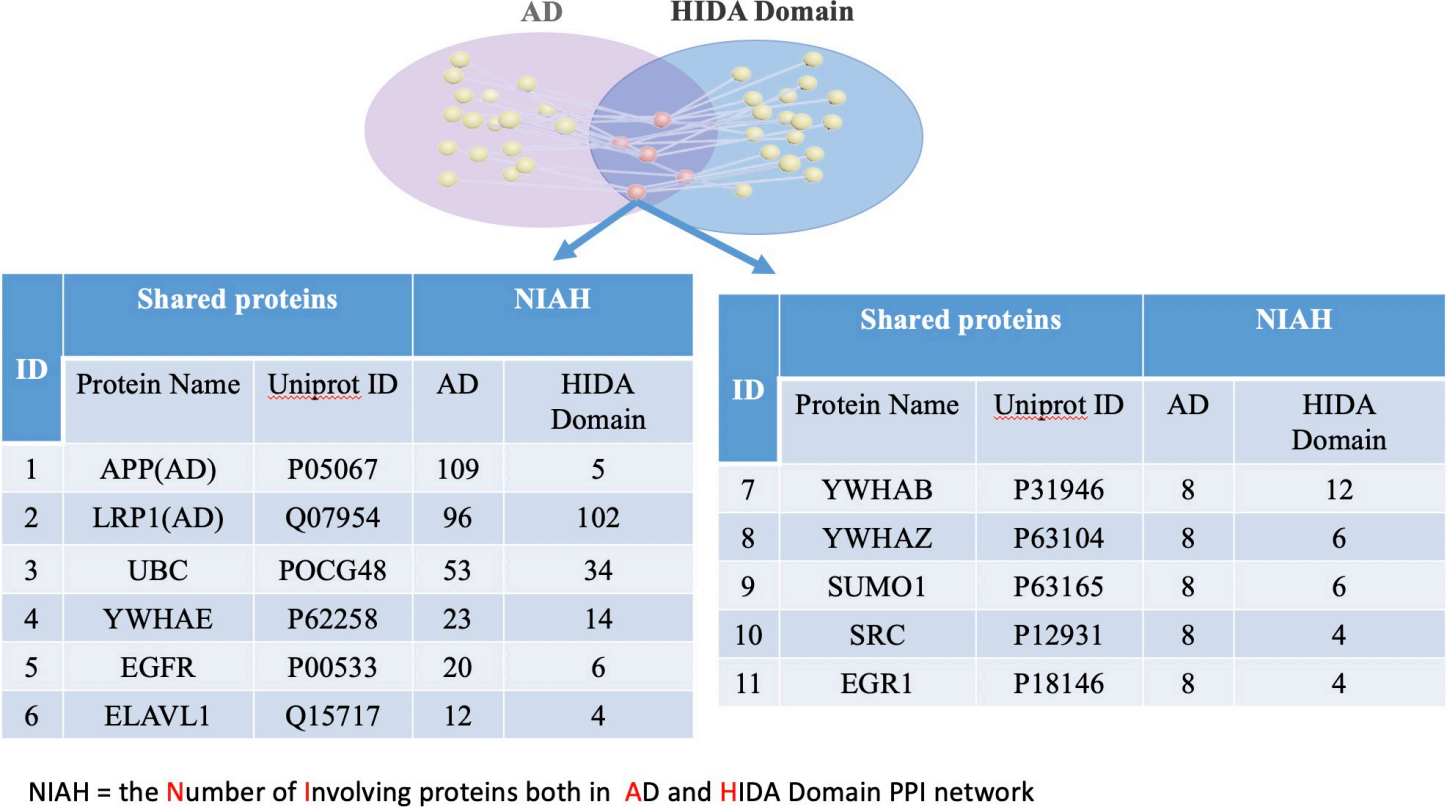
The essential shared proteins between AD and the HIDA Domain. There were 11 proteins that were identified as being essential shared proteins between AD and the HIDA Domain. Proteins in red are essential shared proteins that are unique to the HIDA Domain while those in black were also found to be essential shared proteins between AD, the Affective Domain, and the HIDA Domain.

**Fig 7:**
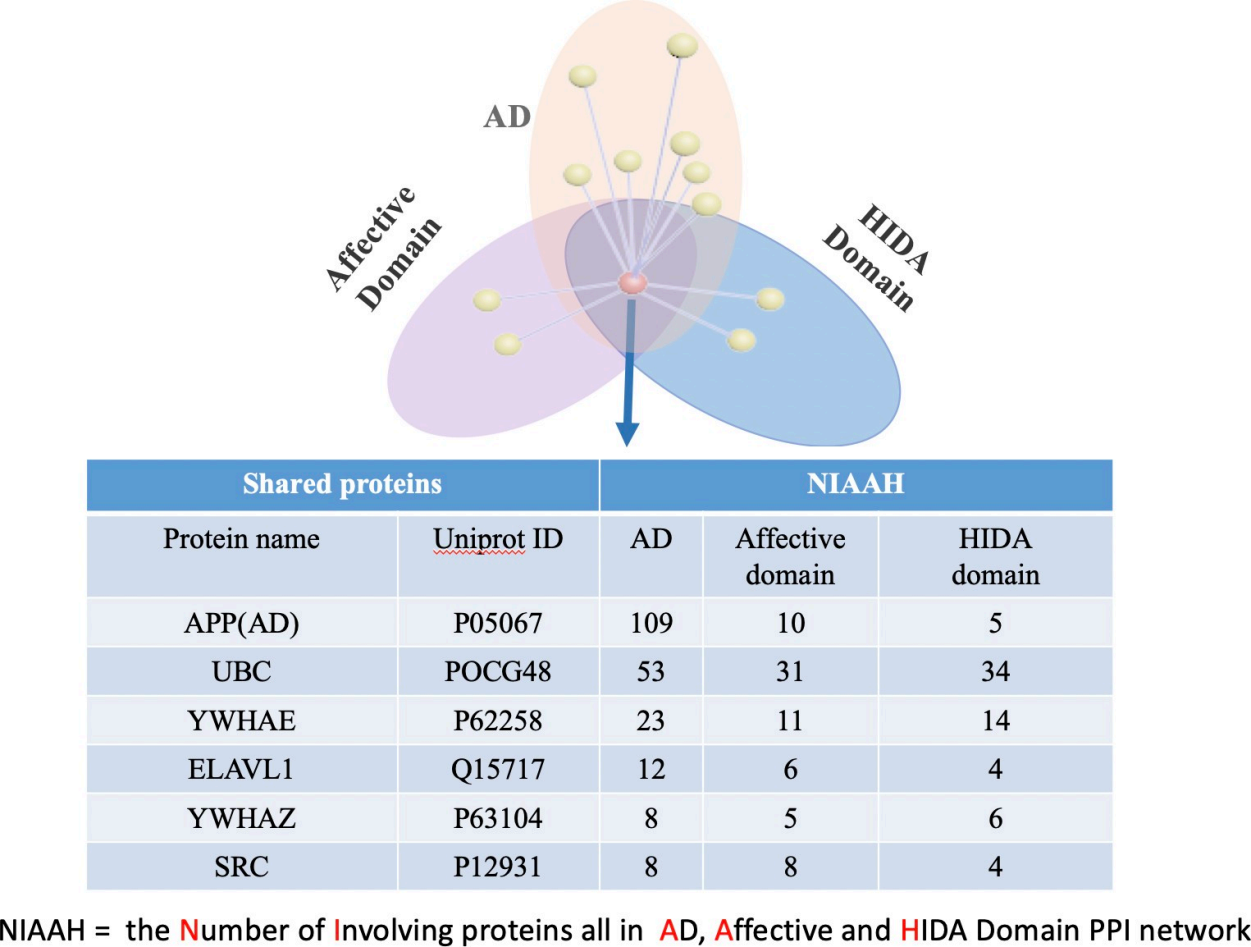
The essential shared proteins among AD and the Affective and HIDA Domains. 6 proteins were identified as being essential shared proteins between AD, the Affective Domain, and the HIDA Domain.

According to the “shared protein-degree centrality” principle, the proteins with high node degree, defined as node degree greater than the mean, and shared among the domains and AD are counted as essential shared proteins. We identified eight unique essential shared proteins between the Affective Domains and AD, (**Fig 5**), five unique essential shared proteins between the HIDA Domain and AD (**Fig 6**), and eight overlapping essential shared proteins between the Affective Domain, HIDA Domain and AD (**Fig 7).**

The mean node degree connecting AD and Affective Domain proteins was six and five, respectively. For each protein in **Fig 5**, the node degree connecting AD and Affective Domain proteins were equal to or greater than the mean node degree, thus, RHOA, APP, UBC, YWHAE, ELAVL1, GRB2, PIK3R1, SRC, HSPA4, YWHAZ, HSP90AA1, GSK3beta, PRKCZ, and FYN were found to be essential shared proteins, in which RHOA and APP are also causative genes related AD, and GSK3beta and PRKCZ are causative genes related to Affective Domain symptoms.

For each protein in **Fig 6**, the mean of degree centrality for each protein was eight and four between the AD and HIDA PPI networks, respectively. The degree centrality for each protein listed in the **Fig 5** is equal to or greater than the mean of degree centrality, thus, APP, LRP1, UBC, YWHAE, EGFR, ELAVL1, YWHAB, YWHAZ, SUMO1, SRC, and EGR1 are essential shared proteins, in which APP and LRP1 are also causative genes related AD.

The mean number linking AD, Affective Domain, and HIDA Domain proteins are eight, eight, and four, respectively. For each protein in **Fig 7**, the mean number linking AD, Affective Domain, and HIDA Domain proteins are equal to or greater than the mean degree centrality, thus, APP, UBC, YWHAE, ELAVL1, YWHAZ and SRC proteins are essential shared proteins across AD and both BPSD domains.

### Functional enrichment analysis

GO term and KEGG pathway enrichment analyses were performed based on the PPI networks of shared proteins between AD and each BPSD Domain. The GO term analysis included three categories: biological processes (BP), molecular functions (MF), and cellular components (CC). **Tables 1** and **2** describe five notable GO terms for BP, MF, and CC as well as five notable KEGG pathways that are enriched between AD and the Affective Domain. These notable terms include Vascular Endothelial Growth Factor (VEGF) signaling, chaperone binding, dendritic spine, and NF-kappa-B signaling in BP, MF, CC, and KEGG, respectively. Between AD and the HIDA domain, there were notable enrichments for Ephrin Receptor signaling, SNAP receptor activity, SNARE complex, and Amyotrophic Lateral Sclerosis (ALS) signaling in BP, MF, CC, and KEGG, respectively (**Tables 3 and 4).** Additional functional enrichment analyses are shown in **S4** and **S5 Tables.**

**Table 1:** Five notable significantly enriched GO terms between AD and the affective domain.

	Terms	Names of shared essential proteins	P value
Biological Process	Vascular endothelial growth factor receptor signaling pathway	SRC, PIK3R1, FYN, RHOA(AD), HSP90AA1	1.62E-13
	Ras protein signal transduction	GRB2	1.44E-08
	Response to cytokine		1.28E-06
	Response to stress		1.89E-06
	Negative regulation of reactive oxygen species metabolic process	HSP90AA1	1.50E-05
Cell Colocalization	Protein complex	HSP90AA1	1.62E-27
	Postsynapse	GSK3beta(AF)	8.93E-08
	Dendritic spine	APP(AD)	9.95E-08
	Histone deacetylase complex		2.76E-07
	Nuclear chromosome, telomeric region		8.81E-06
Molecular Function	Chaperone binding		6.62E-09
	Bisphosphate 3-kinase activity	GRB2, PIK3R1	7.40E-08
	Non-membrane spanning protein tyrosine kinase activity	GRB2, SRC, FYN	2.53E-07
	Heat shock protein binding	SRC,FYN	3.85E-06
	Histone acetyltransferase activity		1.74E-05

Five notable significantly enriched GO terms between AD and the Affective Domain. There were 197 significant Biological Processes (BP), 78 Molecular Functions (MF), and 75 Cellular Compartments (CC) identified in the PPI network of shared proteins between AD and Affective Domain symptoms. Five of the most notable pathways in each group that did not show up in the AD and HIDA Domain PPI network are shown above.

**Table 2:** Five notable significantly enriched KEGG pathways between AD and the affective domain.

Terms	Names of shared essential protein	P value
Toll-like receptor signaling pathway	PIK3R1	9.57E-13
GnRH signaling pathway	GRB2, SRC	4.00E-12
Ras signaling pathway	RHOA(AD), GRB2, PIK3R1	1.32E-11
AMPK signaling pathway	ELAVL1, PIK3R1	6.65E-10
NF-kappa B signaling pathway		1.49E-10

Five notable significantly enriched KEGG pathways between AD and the Affective Domain. There were 66 significant KEGG pathways identified in the PPI network of shared proteins between AD and Affective Domain symptoms. Five of the most notable pathways in each group that did not show up in the AD and HIDA Domain PPI network are shown above.

**Table 3:** Five notable significantly enriched GO terms between AD and the HIDA domain.

	Terms	Names of shared essential proteins	P value
Biological Process	Ephrin receptor signaling pathway	SRC	2.48E-09
	Protein sumoylation	SUMO1	9.56E-08
	Vesicle-mediated transport		1.10E-07
	Response to ethanol		3.98E-07
	Toxin transport		1.65E-06
Cellular Component	Endocytic vesicle membrane	LRP1(AD)	3.51E-09
	Proteasome complex		6.87E-09
	SNARE complex		8.94E-08
	Cytoplasmic vesicle membrane	YWHAE, YWHAB, YWHAZ	1.99E-06
	Terminal bouton	APP(AD)	4.20E-06
Molecular Function	Cadherin binding involved in cell-cell adhesion	YWHAE, EGFR, YWHAB, YWHAZ, SRC	1.18E-17
	SNARE binding		3.54E-08
	SNAP receptor activity		4.12E-07
	Nitric-oxide synthase regulator activity	EGFR	6.77E-06
	Receptor tyrosine kinase binding		9.40E-06

Five notable significantly enriched GO terms between AD and the HIDA Domain. There were 140 significant Biological Processes (BP), 57 Molecular Functions (MF), and 60 Cellular Compartments (CC) identified in the PPI network of shared proteins between AD and HIDA Domain symptoms. Five of the most notable pathways in each group that did not show up in the AD and Affective Domain PPI network are shown above.

**Table 4:** Five notable significantly enriched KEGG pathways between AD and the HIDA domain.

Terms	Names of shared essential proteins	P value
Amphetamine addiction		5.76E-08
Notch signaling pathway		2.87E-07
Cholinergic synapse		2.83E-06
Glutamatergic synapse		4.65E-06
Amyotrophic Lateral Sclerosis (ALS)		1.60E-05

Five notable significantly enriched KEGG pathways between AD and the HIDA Domain. There were 66 significant KEGG pathways identified in the PPI network of shared proteins between AD and HIDA Domain symptoms. Five of the most notable pathways in each group that did not show up in the AD and Affective Domain PPI network are shown above.

## Discussion

In this study, we used publicly available, bioinformatic databases to investigate the possible interactions between AD and the Affective and HIDA Domains and discovered a specific set of shared essential proteins that may be involved in each BPSD domain. In particular, to derive these shared essential proteins, we selected candidate genes from OMIM associated with AD and commonly co-occurring symptoms within each domain: anxiety and depression for the Affective Domain, and hyperactivity, impulsivity, disinhibition, and aggression for the HIDA Domain. Then, we established PPI networks, where common points of convergence between lists were detected using a DBruteForce algorithm, and essential proteins were identified using the “shared protein-degree centrality” principle. These identified “hubs” present a set of proteins that may be adversely affected during AD pathogenesis and contribute specifically to the symptoms within their associated BPSD domain.

The lists of essential shared proteins between AD, the Affective Domain, and the HIDA Domain are inferences based on known protein interactions and thus represent only potentialities that may be central to BPSD pathogenesis. Moreover, use of degree as the sole metric of protein essentiality may result in biased information, although in general, the higher the number of proteins connected between AD and BPSD, the more likely that these proteins drive biological mechanisms and perform physiological functions that are similarly perturbed in AD and BPSD. However, in some cases, high connectivity does not necessarily imply essentiality, and nodes with low degrees might also be relevant despite their relative underconnectedness. In addition, connectivity between proteins is influenced by how often these proteins are interrogated empirically, thus potentially overrepresenting the essentiality of more commonly studied proteins. Finally, some proteins, such as adapter proteins, ubiquitins, and chaperons that bind nonspecifically to a large number of other proteins are more likely to be represented given their promiscuity and multiple protein-interacting motifs. Therefore, it will be important to verify the current analyses with other algorithms that may better account for these known biases. Additionally, rigorous investigation will be necessary to confirm these potential mechanisms. As complex symptoms like BPSD are likely to result from multiple aberrations among many genes/proteins, commonalities between these proteins in terms of pathway or function may be more informative than the individual proteins investigated in isolation. In addition, it is interesting that there are proteins that appear on both lists, which suggest some common driver of neurodegenerative pathology leading to BPSD. Still, the proteins that are unique to one domain seem more likely to be implicated in the generation of specific symptoms. Regardless, the next few sections will help to contextualize the proteins identified here and point out commonalities that may serve as future hypotheses detailing disruption of convergent pathways that cause these symptoms in AD.

### Affective domain

The unique proteins implicated as essential to the Affective Domain broadly fall into two categories functionally, either as chaperones (HSPA4, HSP90AA1) or proteins involved in phosphorylation (FYN, GRB2, GSK3beta, PI3KR1, PRKCZ, RHOA). Though chaperones are likely to be influenced by phosphorylation, and many client proteins for heat shock proteins are kinases[27, 28], both sets of proteins may independently or synergistically affect other pathological mechanisms that ultimately lead to affective symptoms in AD.

One point of convergence among these proteins concerns their regulation of hyperphosphorylated tau (p-tau), an important protein involved in AD pathogenesis. In addition to amyloid-beta (Abeta), p-tau aggregation is a hallmark of AD pathogenesis, with p-tau forming intracellular tangles that, along with Abeta are often used to corroborate post-mortem pathological assessments with ante-mortem clinical diagnosis of AD. In AD, abnormal tau starts developing due to several post-translational modifications that lead to p-tau forming oligomers, paired helical filaments, and straight filaments[29–32]. Though the exact mechanism whereby tau leads to AD pathogenesis is far from clear, tau has been shown to inhibit microtubule formation[33], impair axonal transport[34, 35], and promote neuronal toxicity[36]. One of the earliest steps in AD pathogenesis is the hyperphosphylation of tau at multiple residues, leading to disruption of tau at microtubules and relocation of tau to somatodendritic compartments, influencing synaptic dysfunction[32, 33, 37, 38].

While there is still some debate as to whether the synaptic dysfunction from p-tau stems from pre- or postsynaptic mechanisms[37], it has been well-established that synapse loss is one of the strongest correlates of cognitive dysfunction in AD[39–42]. The loss of synapses is especially intriguing in terms of affective symptoms in AD, as synapse loss in key limbic areas is also correlated with affective symptoms, especially for depression[43–46]. Morphologically, spine loss in the hippocampus and medial prefrontal cortex represents one of the most common pathological sequelae of chronic stress leading to depression[46, 47]. In addition, impairments in proper BDNF expression are similarly noted in both depression and AD[48]. Speculatively, differential post-translational modifications of tau could result in divergent effects on synaptic function in a cell type- and brain area-specific manner, leading to affective symptoms when the balance of certain tau modifications are achieved. With the complex regulation of tau through multiple post-translational modification sites, alternative splicing of 6 isoforms in human brains, and multiple structural confirmations[38, 49, 50], tau does not lack in appropriate complexity for leading to the heterogeneous presentation of symptoms throughout the course of AD.

The longest tau isoform has close to 80 phosphorylation sites, and of these, at least 20 have been implicated in functional alterations of tau[51]. Though many kinases have been associated with tau, one of the most investigated of them is GSK3beta, which phosphorylates tau at multiple residues, resulting in complex functional outcomes on a molecular and cellular basis[52, 53]. GSK3beta’s involvement in AD is further supported by the fact that AD patients have elevated levels of GSK3beta [54, 55] and that familial AD-associated protein PS1 helps localize the kinase to tau[56]. GSK3beta is perhaps the most promiscuous kinase in mammals[57] and so, unsurprisingly, has been associated with the pathogenesis of affective disorders[58, 59]. Notably, many of the treatments for depression have inhibitory effects on GSK3beta, chronic stress increases GSK3beta, and small molecule inhibitors of GSK3beta have been shown to decrease depression-like behavior in pre-clinical models[58]. Highlighting the potential importance of GSK3beta in the Affective Domain of AD, two other kinases that were implicated from our PPI analysis, PI3K and PKC, regulate GSK3beta and have been shown to influence its ability to hyperphosphorylate tau[60, 61]. Thus, GSK3beta represents a possible target for Affective Domain symptoms, and it may be prudent to investigate these symptoms in clinical trials that are testing the effects of GSK3beta inhibitors on cognition in AD. However, if improvement in affective symptoms were realized, further investigation would be needed to determine if inhibition of GSK3beta caused improvements via reduction in p-tau or other mechanisms.

In addition to kinase regulation of p-tau, heat shock proteins have also been implicated in regulating p-tau stabilization and function in AD[62, 63]. Heat shock proteins are induced by cellular stress and are important for protein folding and proteosomal degradation[63]. Of the eight unique proteins identified in our PPI analysis, two are heat shock proteins. HSPA4 is in the HSP110 family and acts as a nucleotide exchange factor for HSP70, aiding in its function[64], and HSP90AA1 is the stress-inducible isoform of HSP90[65]. Both HSP70 and HSP90 have been implicated in the regulation of p-tau, though interestingly, in divergent ways. While HSP70 has been shown to increase p-tau degradation and stabilize tau binding to microtubules[66–68], HSP90 may actually maintain oligomeric p-tau levels, which are quite pathogenic[69, 70]. Thus, the HSP70/HSP90 balance may be a key factor in the regulation of p-tau and resulting affective symptoms. Making matters more interesting, the peptidyl-prolyl isomerase FKBP51 is an interaction partner for HSP90 that seems to facilitate the preservative function of the chaperone on oligomeric p-tau[70]. The point of intrigue comes from the well-described role for FKBP51 in regulation of glucocorticoid receptor localization and function, underlying FKBP51’s prominent role in the manifestation of pathological behavior to chronic, unpredictable, or extreme stress[71]. Though the role for FKBP51 in Affective Domain symptoms remains speculative, the hypothesis that imbalance between HSP70/HSP90 promotes p-tau is intriguing, especially if alterations in subcellular distributions lead to increased p-tau-dependent dysregulation of synaptic activity.

Though many of the proteins identified in this PPI analysis are connected to increased p-tau, a question remains as to how this elevation leads to affective symptoms. As mentioned, one hypothesis is that hyperphosphorylation of tau results in aberrant localization to dendritic compartments, thus leading to postsynaptic dysfunction in key limbic areas affected during AD pathogenesis, such as the medial prefrontal cortex and hippocampus[72]. Interestingly, a few of the proteins identified in our PPI analysis may provide insight into how this postsynaptic dysregulation may be achieved. FYN is a cytosolic tyrosine kinase that was found to be central for oligomeric Abeta-induced synaptic loss and dysfunction[73]. In addition, overexpression of FYN in an AD mouse model accelerated synapse loss and cognitive deficits, with loss of FYN reversing these AD-related sequelae[74, 75]. While the mechanism of synaptic dysfunction was initially unclear, it was discovered that dendritic tau helps localize FYN to the synapse and that loss of functional tau inhibited Abeta-induced synaptic dysfunction[76]. Ultimately, FYN has been shown to regulate the expression of important synaptic plasticity proteins, most notably NMDAR and PSD95, though differential phosphorylation patterns of tau have been shown to promote or ameliorate synaptic dysfunction by this mechanism[76, 77]. Interestingly, in a mouse model of chronic stress, cognitive and affective disorder-like behavior necessitated tau expression to develop. In this same study, glucocorticoids were found to increase p-tau and promote dendritic mislocalization of tau, and FYN was similarly upregulated at the synapse of mice subjected to chronic stress but only if tau expression remained intact, correlating with a change in NMDAR synaptic expression[78]. Though implications for FYN in affective disorders have been less well characterized, there is a report of FYN polymorphisms being associated with trait anxiety[79].

In addition to FYN directly affecting synaptic function via phosphorylation of key postsynaptic density proteins, FYN also regulates RHOA[80, 81], an important regulator of spine morphology and dendritic complexity[82, 83] as well as being another protein identified by our PPI analysis for the Affective Domain. However, while RHOA co-localizes with p-tau[84] and has been shown to influence phosphorylation of tau at certain residues via ROCK[85], it is unclear if a reverse relationship exists whereby p-tau leads to RHOA-dependent alterations of the synapse. A mechanism whereby p-tau would affect RHOA signaling is especially intriguing, as chronic stress has been shown to negatively affect postsynaptic morphology in a RHOA-dependent manner in both medium spiny neurons[83] and pyramidal cells[82], resulting in affective symptoms.

In summary, the eight unique proteins identified by our PPI analysis suggest that regulation of p-tau may be highly involved in the Affective Domain. The increase in p-tau species could lead to greater mislocalization of tau to postsynaptic compartments, which may lead to significant synaptic dysregulation. Plausibly, both FYN and RHOA may be mediators of this synaptic dysfunction through alterations in key synaptic proteins and dendritic structure.

### HIDA domain

Unlike the Affective Domain, where proteins can be neatly sorted into two categories, the unique proteins in the HIDA Domain have relatively diverse functions. Specifically, these proteins are Low Density Lipoprotein Receptor-Related Protein 1 (LRP1), a cholesterol receptor that mediates endocytosis and binds to over 40 ligands[86], Epidermal Growth Factor Receptor (EGFR), a receptor tyrosine kinase in the neuregulin/ERBB family[87], 14-3-3beta (YWHAB), a phosphoserine/phosphotyrosine binding protein of the 14-3-3 family that interacts with a wide-range of other proteins to facilitate protein interactions[88], Small Ubiquitin Related Modifier 1 (SUMO1), a ubiquitin-like post-translational modification that affects a broad range of protein functions[89], and Early Growth Receptor 1 (EGR1), a transcription factor that represents an intermediate-early gene marking neuronal activation and synaptic plasticity[90]. All five of these proteins have been implicated in AD pathogenesis: LRP1 is a receptor that interacts with Abeta and APOE, which may influence LRP1’s ability to aid in Abeta clearance[86]; EGFR polymorphisms were found to be inversely proportional to AD risk in a Han Chinese cohort[91]; 14-3-3 can be found around Abeta plaques and facilitates GSKbeta-mediated phosphorylation of tau[92]; SUMO1 expression is altered in cortices of AD patients[93]; and EGR1 inhibition leads to decreased Abeta and p-tau while reversing cognitive deficits in 3xTg AD mice[94]. However, the links between these proteins and HIDA Domain symptoms are sparse. Despite this paucity of direct evidence in HIDA Domain symptoms, some points of convergence in perturbed pathways may suggest how these proteins could influence the development of these symptoms in AD.

To conceptualize how these proteins may lead to HIDA Domain symptoms, understanding the broad mechanisms that underlie these symptoms is helpful. Though at first hyperactivity, impulsivity, disinhibition, and aggression may seem like distinctly different entities, they share a common conceptual framework in a lack of inhibitory control over one’s actions. Unsurprisingly, some common circuitries are perturbed in the development of these symptom, most notably key corticostriatal pathways[95–97].

For instance, aggression is often partitioned into impulsive/reactive aggression and instrumental/proactive aggression, of which the former is most likely to occur in AD[97, 98]. Over many human imaging and animal studies, three important circuits that mediate impulsive aggression seem to emerge: 1) areas that support aggressive impulses, including the amygdala, periaqueductal gray, anteroventral medial hypothalamus, lateral septum, and medial preoptic nucleus, 2) cortical decision-making centers that evaluate the consequences of an action and regulate emotion, such as the orbitofrontal prefrontal cortex (oPFC), ventromedial prefrontal cortex (vmPFC), and anterior cingulate cortex (ACC), and 3) striatal areas mediating response inhibition and encoding the rewarding and reinforcing aspects of aggressive acts[96, 97, 99–101]. In aggression, the cortical decision-making circuitry has an inhibitory influence on the impulses for aggressive behavior, though admittedly this is an overly simplistic description of a complex and nuanced system[97].

When compared to aggression, the circuitry for impulsivity is surprisingly similar: impulsivity can generally be increased with activation of the ventral tegmental area (VTA) to the ventral striatal circuit and is facilitated by reduced activation or lesioning of numerous frontal cortical areas, such as the vmPFC, oPFC, and ACC[95, 99]. In addition, areas like the ventral hippocampus and amygdala can modulate behavior in certain types of impulsivity[95]. For motor response inhibition, which may be associated with agitation and hyperexcitability in AD patients, the ACC, subthalamic nucleus, and certain pre-motor cortical areas, among others, are implicated[95, 102]. Again, while highly simplified, the HIDA Domain seems to be mechanistically united in its dependence on corticostriatal balance.

In addition to corticostriatal involvement, HIDA Domain symptoms have been shown to be influenced by monoaminergic modulation, namely by serotonin, norepinephrine, and dopamine[95, 96, 101]. Monoaminergic regulation of multiple behaviors, including those in the HIDA Domain, is achieved through broad release of these neurotransmitters in almost every area of the brain, especially among neocortical and limbic areas[103, 104]. In general, reduction in signaling of these three molecules leads to a greater burden of HIDA Domain symptoms, especially when affecting cortical and striatal areas[95, 96, 101]. In addition, multiple monoaminergic signaling genes have been implicated in increased risk of impulsivity, aggression, or disinhibition, including *Drd1*[105], *Drd2*[106, 107], *Drd4*[106, 107], *5htr1a*[108], *5htr1b*[109–111], *5htr2a*[112–114], *5htr2b*[115], *Dat1*[116], *5htt*[117–119], *Maoa*[120–122], *Comt*[116, 123, 124], and *Tph2*[125]. Thus, reductions in monoaminergic regulation of corticostriatal pathways represents another complementary mechanism whereby HIDA Domain symptoms may appear.

In AD, some of the first areas to show neuropathology, degeneration, and dysfunction include the locus coreuleus (LC), the main center for noradrenergic signaling, and the dorsal raphe nucleus (DRN), a main center that provides serotonergic innervation for limbic and cortical areas[103, 126, 127]. In addition, some degeneration of the VTA, the main output center for the mesolimbic dopamine circuit, exists and has a functional impact on AD symptoms, though to a lesser extent than the LC and DRN[127–129]. Loss of monoaminergic innervation to corticostriatal circuits may therefore represent an early mechanism that leads to HIDA Domain symptoms. In contrast, while the transtentorial cortical region tends to degenerate early in AD, the neocortical regions seem to develop AD-related pathology slightly later in disease progression and radiate from the ventral to dorsal cortices[103, 130]. There is also some evidence that the striatum undergoes significant degeneration with AD[131]. Thus, selective loss of corticostriatal pathways may underlie a relatively late mechanism of HIDA Domain pathogenesis.

Perhaps the most straightforward link between these proteins and the HIDA Domain would be in their ability to influence cell death. LRP1 promotes survival via an Akt-mediated mechanism[132], EGFR protects cells from neurotoxicity through Akt downstream of PS1[133], 14-3-3 is broadly involved in neuronal differentiation and survival[92], inhibition of SUMO1 decreases neuroprotection to ischemia[134], and EGR1 promotes maturation and survival of dentate granule cells[135]. Alterations in these proteins may cause degeneration of cells in monoaminergic or cortical areas that regulate corticostriatal circuitry during AD pathogenesis, thus leading to presentation of HIDA Domain symptoms. While possible, synaptic dysfunction precedes cell death in AD pathogenesis, and it is more likely that HIDA Domain symptoms are present before a large burden of cell death occurs. In addition, it is unclear why these particular proteins would be implicated in being neuroprotective for the HIDA Domain, as few of them have unique ties to cortical or monoaminergic areas, though EGFR and 14-3-3 have been implicated in dopaminergic cell survival[136–139].

A more intriguing link between these proteins involves their ability to regulate axon remodeling and presynaptic function. Though synapse loss is generally agreed to be the best predictor of symptom progression in AD, axonal pathology and dysregulation are some of the earliest events in AD pathogenesis, often being detectable before plaque formation[34, 140, 141]. Specifically, axonal degeneration is typified by axonal swelling, demyelination, axonal atrophy, loss of presynaptic markers, deficits in axonal transport mechanisms, and eventual dying back neuropathy[34, 140, 141]. In addition, presynaptic dysfunction occurs in AD and may further contribute to symptoms in addition to morphological degeneration of axons[142]. Presynaptic dysfunction and axonal degeneration in cortico-cortical, corticostriatal, and monoaminergic axons may thus represent a mechanism whereby HIDA Domain symptoms develop. This may especially be true for monoaminergic axons, which are poorly myelinated and travel far distances, thus making them particularly susceptible to small aberrations in the cellular environment and increasing their dependence on efficient axonal transport[103].

In addition, it has been suggested that the areas of earliest AD pathology are also some of the most plastic structurally, which may be necessitated by the constant remodeling that is required to encode experiences and make decisions in dynamic contexts. Accordingly, it has been suggested that AD occurs as a process of continuous synaptic organization gone awry, leading to dedifferentiation and loss of synapses[143]. In support of AD being a disease of dysregulated structural plasticity, abnormal sprouting at both post- and presynaptic areas is an early hallmark of AD pathogenesis[143].

Thus, it is intriguing that the five unique proteins identified by our PPI analysis are involved in presynaptic function and axon maintenance. For instance, LRP1 is necessary for axonal extension mediated by lipoproteins, and its function can be enhanced by non-pathogenic ApoE3 but not pathogenic ApoE4[144], a process which is dependent on MAPK and intracellular calcium[145]. In addition, LRP1 agonism promotes sensory axon sprouting and regeneration after spinal cord injury[146], and axonal regeneration through the urokinase-plasminogen activator is modulated by LRP1 independent of integrin beta-1[147]. Finally, LRP1 was shown to be necessary for metallothienen-II’s ability to overcome microglial upregulation of TNF-alpha and thus promote axon growth[148]. As LRP1 is a receptor allowing for endocytosis of multiple lipoproteins that can integrate into the plasma membrane, it may be an important regulator of axonal structural plasticity by allowing for the necessary plasma membrane substrates to reach their presynaptic location.

EGFR has also been shown to impact axonal structure. Specifically, EGFR is best known as being necessary for restriction of axonal regeneration in the CNS mediated by inhibitory substrates, such as myelin and chondroitin sulfate proteoglycans[149–151]. Interestingly, EGFR may not just promote inhibition of axonal regeneration, it may also be essential for maintaining the proper balance between dynamic and static filopodia. Specifically, it was shown that EGFR demonstrates an intrinsic asymmetry during axon outgrowth monitored by live cell imaging, and aberrations in EGFR signaling led to reduced outgrowth dynamics and excessive branch formation[152]. Thus, EGFR may be an important protein mediating the “structural plasticity” hypothesis of AD and could exacerbate connectivity breakdown between corticostriatal or monoaminergic pathways.

14-3-3 Proteins have also been highly implicated in axon regeneration and structural plasticity[88, 153]. In particular, knockdown or inhibition of 14-3-3beta can promote axon and neurite outgrowth[154]. Similar to the dynamic role of EGFR, 14-3-3beta has also been shown to be able to promote the switch from attraction to repulsion of axons induced by Sonic Hedgehog signaling[155]. Interestingly, it was shown that 14-3-3 may interact with p-tau to promote tubulin disruption, thus impacting proper axon maintenance or neurite outgrowth and directly linking this axonal regulation with AD pathogenesis[156].

Unlike the previous three proteins, SUMO1 has been more closely linked to presynaptic signaling mechanisms than axonal maintenance. However, it was shown that SUMO1 attaches to the RNA binding protein La to facilitate its retrograde transport, implicating this post-translational modification in axonal transport[157]. Presynaptically, however, SUMO1 has been shown to be necessary for the localization of several key proteins involved in presynaptic vesicle recruitment and release, including synapsin Ia[158], RIM1-alpha[159], and gephryn[160]. In addition, SUMO1 actively influences presynaptic transmission, as evidenced by SUMO1 mediating decreased presynaptic glutamate release in the AD mouse model Tg2576[93]. Thus, SUMO1 may contribute to deficits in axonal transport as well as reduced presynaptic function in AD.

Finally, despite EGR1’s role as a transcription factor, and thus exerting the bulk of its actions in the nucleus, it has also been implicated in influencing axon growth. Specifically, inhibition of EGR1 prevents the ability of NGF to increase neurite outgrowth in a novel pathway whereby EGR1 binding to c-Jun promotes NGF’s downstream effects[161]. In addition, PACAP also depends on EGR1 for its ability to promote neuritogenesis[162]. Lastly, sAPPbeta, the cleavage product of beta-secretase and upstream precursor of Abeta, is able to promote neuritic outgrowth but needs functional EGR1 to promote axon outgrowth, while dendritic outgrowth is independent of intact EGR1[163]. Thus, EGR1 joins the other unique proteins associated with the HIDA Domain in our PPI analysis in its ability to influence the structural plasticity of axons and presynaptic function. Altogether, this neurodegenerative process at presynaptic sites may influence corticostriatal and monoaminergic pathways, thus linking these five proteins identified by our PPI analysis and the HIDA Domain.

## Supporting information

S1 Table Selective genes associated with AD and each BPSD domain. (XLSX)

S2 Table The shared proteins between AD and the affective domain. (XLSX)

S3 Table The shared proteins between AD and the HIDA domain. (XLSX)

S4 Table The enriched GO terms and KEGG pathways between AD and the affective domain. (XLSX)

S5 Table The enriched GO terms and KEGG pathways between AD and the HIDA domain. (XLSX)
